# Family plot: the impact of the endosperm and other extra-embryonic seed tissues on angiosperm zygotic embryogenesis

**DOI:** 10.12688/f1000research.21527.1

**Published:** 2020-01-14

**Authors:** Gwyneth C Ingram

**Affiliations:** 1 Laboratoire Reproduction et Développement des Plantes, University of Lyon, ENS de Lyon, UCB Lyon 1, CNRS, INRAE, Lyon, France

**Keywords:** Seed, Embryo, Endosperm, Communication, Nutrition

## Abstract

The zygotic embryos of angiosperms develop buried deep within seeds and surrounded by two main extra-embryonic tissues: the maternally derived seed coat tissues and the zygotic endosperm. Generally, these tissues are considered to play an important role in nurturing the developing embryo by acting as conduits for maternally derived nutrients. They are also critical for key seed traits (dormancy establishment and control, longevity, and physical resistance) and thus for seed and seedling survival. However, recent studies have highlighted the fact that extra-embryonic tissues in the seed also physically and metabolically limit embryonic development and that unique mechanisms may have evolved to overcome specific developmental and genetic constraints associated with the seed habit in angiosperms. The aim of this review is to illustrate how these studies have begun to reveal the highly complex physical and physiological relationship between extra-embryonic tissues and the developing embryo. Where possible I focus on Arabidopsis because of space constraints, but other systems will be cited where relevant.

## Introduction

The multicellular female gametophyte, situated within the ovule, represents the starting point for angiosperm seed development. In most cases, this is triggered by the arrival of the pollen tube (the male gametophyte, containing two genetically identical sperm cells) and the subsequent quasi-simultaneous fertilization of the egg cell (1n), to give the diploid zygote, and of the homodiploid (2n) central cell, to give the triploid endosperm. These two genetically distinct “siblings” then develop concomitantly within the surrounding maternal tissues. The embryo usually develops almost completely surrounded by the endosperm, although it can directly contact maternal tissues at the micropylar pole ^1^. In many cases, it appears that cytoplasmic contact between maternal tissues, the endosperm, and the embryo are lost soon after fertilization ^2^, meaning that molecular transport between the three tissues must occur via the transcellular pathway, involving transmembrane transport in and out of cells and diffusion across the cell wall (apoplastic) compartment.

As in many angiosperm species, the early development of the endosperm of the model species *Arabidopsis thaliana* initiates with a coenocytic phase, characterized by nuclear divisions in the absence of cytokinesis and by rapid expansion ^3^. During this early phase, the endosperm acts as a major metabolic sink, absorbing nutrients from maternal tissues and sequestering them in its large central vacuole ^4^. Seed size, and thus nutrient storage potential, are determined during this phase. Nutrients are then re-exported from the endosperm for absorption by the embryo ^5– 7^, a process facilitated by the ephemeral basal region of embryo called the suspensor ^8, 9^. Its role in conducting maternal reserves to the embryo has led to parallels being drawn between the angiosperm endosperm and the mammalian placenta ^10– 12^. Intriguingly, and consistent with this analogy, as in the placenta, the angiosperm endosperm, which contains both male and female genomes owing to double fertilization, appears to be a focus for parental conflicts over resource allocation, particularly in outcrossing or partially outcrossing plants ^13^. Specifically, maternal interests are predicted to minimize, or at least equilibrate, nutritional investment between seeds (since mothers are nutrient providers and equally related to all their offspring), whereas paternal interests are predicted to act to maximize maternal nutrient investment. Consistent with this, paternal excess in the endosperm (which can occur, for example, when pollen from tetraploid plants is used to fertilize diploid ovules) tends to increase seed size, whilst maternal interests have the opposite effect. This conflict is proposed to play out, at least partially, at the genomic level through the acquisition of gamete and allele-specific epigenetic regulation (imprinting) ^13– 16^.

Interestingly, in situations of either paternal or maternal excess in the endosperm, specific developmental syndromes, potentially linked to seed size changes, and which are at least in part due to changes in the dosage of imprinted genes (showing preferential expression from either the paternal or the maternal allele), are also observed ^17^. These syndromes frequently lead to reduction or loss of seed viability named the “triploid block”, which can thus cause an immediate post-zygotic hybridization barrier between plants of differing ploidy ^18– 22^. Here some of the mechanisms underlying this phenomenon in *Arabidopsis thaliana* are discussed. Seed development is examined more generally in light of possible parental conflicts, with the aim of shedding new light on key interactions between the developing embryo and surrounding tissues ^23^.

## Coordinating early post-fertilization development: the role of auxin

Maternal interests (which according to kinship theory should restrict resource uptake by the endosperm) are, in part, managed by the repressive activity of a central cell/endosperm-specific variant of Polycomb Repressive Complex 2 (PRC2) called Fertilization Independent Seed (FIS)-PRC2 ^24– 28^. FIS-PRC2 represses the initiation of endosperm proliferation (and thus maternal resource allocation) in the absence of fertilization. To mediate this function in Arabidopsis, FIS-PRC2 has recently been shown to act by repressing genes encoding auxin biosynthetic enzymes, and auxin production in the central cell has been shown to be sufficient to trigger endosperm proliferation and expansion. Thus fertilization, which introduces transcriptionally active copies of these genes carried by sperm cells to the endosperm, can trigger endosperm proliferation ^29^. Intriguingly, auxin efflux from the endosperm has also been shown to be necessary for the post-fertilization differentiation of maternal tissues, which is necessary for efficient resource provision to the developing endosperm ^30^. Furthermore, auxin derived from maternal tissues adjacent to the suspensor, and presumably actively transported to the embryo, appears to be required for early embryonic patterning in Arabidopsis ^1^. Although, in Arabidopsis, direct links between endosperm-derived auxin and embryo development remain elusive, work in maize has led to suggestions that the endosperm auxin maximum could both guide and regulate early embryo growth ^31^.

## The pressure is on: endosperm expansion *versus* embryo establishment

In Arabidopsis, the major endosperm/seed growth phase is driven by expansion of the coenocytic endosperm. Importantly, this early expansion, combined with controlled endosperm elimination (see below), conditions the final size of the embryo by determining the space available for embryo expansion later in seed development (reviewed in ^32– 34^). Early endosperm expansion is likely driven, at least in part, by the accumulation of osmotically active metabolites, including sugars and amino acids, in the central endosperm vacuole ^4, 35, 36^. Consistent with the parental conflict theory, seed growth is also known to be physically constrained by maternal tissues (the seed coat) ^37, 38^. This constraint has recently been shown to involve an active response to the tension that builds up in maternal tissues due to the expansion of the endosperm ^37^. How tension is perceived within the seed coat remains poorly understood. However, it likely regulates growth through modification of specific cell walls within the seed coat, potentially via the degradation of gibberellic acid, a growth-promoting hormone. This model suggests that the internal pressure of the endosperm must be tightly regulated over time in order to achieve optimal seed expansion, since either excessive or insufficient endosperm pressure could lead to growth defects. The activity of invertases, and other enzymes within the endosperm, which cleave sucrose (the main sugar absorbed by the endosperm) into hexoses, thereby lowering the osmotic potential of the endosperm, could play important roles in this regulation ^4, 36, 39– 41^. However, other potentially key processes, such as the regulation of nutrient transporter activity and water movement, remain poorly understood.

In Arabidopsis, the major endosperm/seed growth phase ends with endosperm cellularization, which involves cell wall outgrowth into the endosperm cavity and nuclear partitioning ^42– 44^. Endosperm cellularization and final seed size are tightly linked, with premature cellularization associated with small seed size (as seen in maternal excess situations) and lack of cellularization associated with large seeds (seen in paternal excess situations, and when FIS-PRC2 function is defective) ^36, 45^. One factor underlying this coupling could be the reported link between a decrease in hexose/sucrose ratio in the endosperm and cellularization ^36, 40^. This decrease, although not well understood mechanistically, could explain an observed decrease in the turgor pressure of the endosperm, which should reduce its growth potential ^35^. The cellularization process has also been proposed to increase direct contact between the apoplast of the embryo and that of maternal tissues through the establishment of “apoplastic bridges” ^46^. These have been proposed to enable the embryo to establish itself more efficiently as a sink by effectively bypassing the endosperm. This might explain why the embryos of mutants defective in endosperm cellularization (including those lacking FIS-PRC2 activity) fail to develop past the heart stage ^36^. It could also provide an additional explanation for the link between seed growth and endosperm cellularization since, by increasing embryo sink strength, cellularization could deplete nutrients available for uptake by the endosperm. Promotion of endosperm cellularization by FIS-PRC2 is mediated in part through repression of the endosperm-specific *AGL62* gene, encoding a negative regulator of cellularization ^36, 47, 48^. *AGL62* is not directly imprinted but is regulated by the PHERES1 transcription factor, the expression of which is repressed by FIS-PRC2 ^49, 50^. Interestingly, other PHERES1-binding targets include *HAIKU2* and *MINISEED3* ^38, 51^, which negatively regulate cellularization during early post-fertilization development as well as promoting seed expansion and thus embryo growth ^50^. Endosperm cellularization is also inhibited by auxin, and clear links between retarded cellularization in paternal excess situations and excessive auxin production have recently been established ^52^.

Interestingly, some species appear to have developed alternative strategies allowing the mother plant to bypass the endosperm during resource allocation to the embryo, with notable examples being found in certain members of the Crassulaceae, where the embryonic suspensor forms invasive haustorial extensions within maternal tissues, increasing the surface for direct nutrient transfer ^53^. In Arabidopsis, maternally active signaling peptides produced in the central cell positively regulate the expansion of the suspensor ^54, 55^, which could represent a maternal strategy to increase the ability of young embryos to acquire nutrients from the endosperm. Finally, the transfer of nutrient storage functions to maternal sporophytic tissues (usually in the form of a nucellus-derived perisperm) has also been proposed to represent such a strategy ^56, 57^.

## Making space: endosperm elimination permits embryo growth

Endosperm cellularization, despite potentially opening the way for direct nutrient flow from the maternal apoplast to that of the embryo, also encloses it within a “solid” tissue. Endosperm breakdown would therefore be predicted to promote embryo growth through both physically providing space for embryo expansion and the release/recycling of nutrients stored within the endosperm tissues to fuel embryo growth and storage product accumulation. It might also be expected to reduce endosperm sink strength. In Arabidopsis, the endosperm is a largely ephemeral tissue that breaks down almost completely during seed development. The importance of this process is borne out by the phenotypes associated with loss of function of the endosperm-specific RETARDED GROWTH OF EMBRYO1/ZHOUPI (ZOU) protein. *zou* mutants show a complete lack of endosperm breakdown, leading to a restriction of embryo expansion and a dramatically reduced final embryo size ^58, 59^. To what extent this phenotype is also a consequence of a lack of endosperm nutrient recycling and/or of inappropriate maintenance of endosperm sink strength remains unclear. However, a recent study has linked limited endosperm breakdown around the developing maize embryo to nutrient recycling (Doll *et al*., unpublished data).

The *ZOU* gene is not imprinted but, interestingly, like the repressor of cellularization *AGL62*, has recently shown to be bound by the PHERES1 protein (encoded by an imprinted gene) ^50^. The role of FIS-PRC2 in regulating endosperm breakdown remains difficult to assess, however, since endosperm lacking FIS-PRC2 never cellularize. Strongly increased pressure within *zou* mutant seeds compared to wild-type siblings post-cellularization shows that physical constraints imposed upon the embryo by the endosperm are alleviated by the activity of the ZOU protein to allow embryo expansion ^37, 60^.

## Coming unstuck: resolving a fusional relationship

*zou* mutants are defective not only in endosperm breakdown/embryo growth but also in two other processes that illustrate graphically the degree to which zygotic embryogenesis is influenced by the surrounding endosperm tissues: namely embryo cuticle biogenesis ^61^ and the physical separation of the embryo from the surrounding endosperm ^62^. In Arabidopsis, ** the formation of an intact embryonic cuticle is critical for embryo survival at germination. However, cuticle integrity is established early in seed development, whilst the embryo is still embedded within the endosperm ^63^. The fact that cuticle reinforcement post-germination is strongly influenced by environmental cues ^64– 67^ raises the question of how cuticle integrity is monitored prior to embryo germination. It also raises important questions regarding the properties and functions of the embryonic cuticle, which appears to be permeable to small hydrophilic molecules, consistent with the importance of the embryonic surface in the uptake of nutrients from the endosperm ^63, 68^. The cuticle could thus act as a molecular “filter”, the properties of which appear to vary over the embryonic surface ^68^.

Recent work suggests that the close proximity of the concomitantly developing embryo and endosperm could indeed hinder the development of the embryonic cuticle, a problem which has been overcome through the recruitment of an integrity-monitoring pathway involving a molecular dialogue between the developing embryo and surrounding endosperm tissues ^69, 70^. This dialogue has very recently been shown to depend on proteins produced in both tissues and on the bidirectional diffusion of an embryo-derived, progressively matured peptide (TWISTED SEED1) ^71^, through cuticle “gaps” ^63^ (Doll *et al*., unpublished data). This molecular exchange is necessary to ensure the filling of “gaps” in the embryonic cuticle, but not, apparently, for cuticle biosynthesis *per se*, and triggers cytoplasmic and transcriptional responses similar to those triggered by abiotic and biotic stress ^63^. The expression of the endosperm-specific component in this dialogue, a subtilisin protease, depends upon the function of ZOU. Importantly, this mechanism, depending as it does upon endosperm-specific components, is likely not required during somatic embryogenesis, the process via which embryogenesis occurs through the reprogramming of single somatic cells. Indeed, somatic embryos, which are not surrounded by endosperm and thus potentially perceive other environmental cues, appear able to form an intact cuticle in the absence of endosperm tissues in several species (reviewed in 72).

Although the cuticles of juxtaposed organs prevent post-genital organ fusion post-germination in plants (reviewed in 73), the formation of the embryonic cuticle is not sufficient to ensure the physical separation necessary for the invasive growth of the embryo through surrounding endosperm cells that occurs during seed development. A recent study has revealed that this process requires an additional modification of the embryo–endosperm interface which, in Arabidopsis, takes the form of a glycoprotein-rich “sheath” that is necessary for the embryo to separate from, and slide past, neighboring endosperm tissues ^62^. Although the material that composes the sheath is deposited on the embryo surface (outside the cuticle), it originates in the endosperm, and its production depends upon ZOU, and more particularly the KERBEROS peptide, whose production is ZOU dependent. How the presence of the sheath affects other processes, such as apoplastic nutrient transfer to the embryo, again remains very poorly understood. However, active secretion of glycoprotein-like matrices has been observed at the endosperm–embryo interface of other seeds including those of *Solanum* species ^74^, suggesting that this process may be widespread in angiosperms. Interestingly, sheath deposition at the embryo surface depends on the signaling pathway involved in cuticle integrity monitoring (described above), again indicating a complex dialogue between the embryo and endosperm ^62^.

## Conclusion

The word “altruistic” has frequently been employed to describe the relationship between the angiosperm endosperm and its “sibling”, the zygotic embryo ^55, 75^. Intriguingly, some recent research even suggests that the relatedness of the endosperm to the embryo can influence resource redistribution ^76^. Nonetheless, developing within the endosperm imposes significant physical and metabolic constraints upon the embryo, which have the potential to impede or even to prevent its development entirely ^20, 34, 77^. Some of these constraints arise from the need of the embryo, originating from a single cell within the multicellular female gametophyte, to individualize itself and generate a functional surface prior to germination and exposure to environmental constraints. One of the advantages attributed to double fertilization is the coordination of endosperm development with that of the embryo, thus minimizing resource wastage ^78^. However, forcing the concomitant development of two structures within the confines of the seed coat may have rendered the ability of the embryo to physically and chemically separate from surrounding tissues very difficult, necessitating the acquisition of novel molecular dialogues with surrounding tissues that could allow the embryo to distinguish “inside” from “outside”.

Another profound effect of double fertilization was that the introduction of a male genome into the nutrient-storing tissue of the seed opened the door to parental conflicts over resource allocation. These conflicts, played out principally in the endosperm and mediated, in part, by genomic imprinting, have been proposed to be a key driver in rapid angiosperm speciation since they underlie rapidly established post-zygotic hybridization barriers. Key regulators involved in endosperm development (including auxin biosynthetic enzymes/transporters necessary for endosperm proliferation and seed coat differentiation, factors regulating endosperm cellularization and subsequent breakdown, and, indeed, factors influencing the formation of the embryo surface) appear to have been subsumed directly, or indirectly, into genetic networks acting downstream of imprinted genes. Their analysis has helped to pinpoint critical processes implicated in controlling the physical and nutritional relationships between seed tissues.

Despite recent advances in seed biology, however, remarkable voids in our understanding of fundamental processes remain. These include questions as simple as how the influx of water and nutrients into seeds is regulated (both at the level of transporters and apoplastic interfaces) and how the physical properties of different compartments (turgor pressure and cell wall stiffness) are controlled. The complex structure of the seed and the fact that the embryo is buried deep within it have contributed to the slow rate of progress in answering these questions. However, recent advances in spatially resolved metabolite analysis and imaging should provide a considerable boost to ongoing research.
